# Native plants fare better against an introduced competitor with native microbes and lower nitrogen availability

**DOI:** 10.1093/aobpla/plx004

**Published:** 2017-01-25

**Authors:** W. Gaya Shivega, Laura Aldrich-Wolfe

**Affiliations:** Department of Biology, Concordia College, Moorhead, MN 56562, USA

**Keywords:** *Andropogon gerardii*, arbuscular mycorrhizal fungi, *Carduus acanthoides*, nitrogen availability, *Oligoneuron rigidum*, plant competition, plant–soil feedback, soil microbial community, tallgrass prairie

## Abstract

While the soil environment is generally acknowledged as playing a role in plant competition, the relative importance of soil resources and soil microbes in determining outcomes of competition between native and exotic plants has rarely been tested. Resilience of plant communities to invasion by exotic species may depend on the extent to which native and exotic plant performance are mediated by abiotic and biotic components of the soil. We used a greenhouse experiment to compare performance of two native prairie plant species and one exotic species, when grown in intraspecific competition and when each native was grown in interspecific competition with the exotic species, in the presence and absence of a native prairie soil community, and when nitrogen availability was elevated or was maintained at native prairie levels. We found that elevated nitrogen availability was beneficial to the exotic species and had no effect on or was detrimental to the native plant species, that the native microbial community was beneficial to the native plant species and either had no effect or was detrimental to the exotic species and that intraspecific competition was stronger than interspecific competition for the exotic plant species and vice versa for the natives. Our results demonstrate that soil nitrogen availability and the soil microbial community can mediate the strength of competition between native and exotic plant species. We found no evidence for native microbes enhancing the performance of the exotic plant species. Instead, loss of the native soil microbial community appears to reinforce the negative effects of elevated N on native plant communities and its benefits to exotic invasive species. Resilience of plant communities to invasion by exotic plant species is facilitated by the presence of an intact native soil microbial community and weakened by anthropogenic inputs of nitrogen.

## Teaser

Success of plant invasions may depend on soil resources. In the greenhouse, two native tallgrass prairie species and an exotic invasive exhibited strong differences in performance between soils with and without native microbes and with differing nitrogen availability. Increased nitrogen availability only benefitted the exotic, while native microbes only benefitted the natives. The microbial benefit to native plant growth disappeared under high soil nitrogen, while the negative effect of elevated nitrogen on survival of one of the natives was ameliorated by the presence of the exotic. Nitrogen and soil microbes interact to affect the strength of competition in plant invasions.

## Introduction

Invasion of native plant communities by exotic species is pervasive worldwide, an important contributor to loss of biodiversity both directly through competition with native plant species and indirectly by replacing the plant resources on which specialists at other trophic levels depend, and results in the global homogenization of plant communities (Vitousek *et al.* 1997; Stohlgren *et al.* 2011). Invasions can be facilitated by a variety of anthropogenic factors. In addition to dramatically increasing the dispersal rate of exotic species, humans also facilitate invasions through habitat fragmentation, land use change, global climate disruption and elevating nitrogen (N) availability (Vitousek *et al.* 1997; Davis *et al.* 2000; Suding *et al.* 2005). Soil N availability in many plant communities has been elevated directly by N deposition (Galloway *et al.* 2003) and also indirectly through accelerated rates of mineralization in soils experiencing warmer temperatures in response to anthropogenic climate change (Rowe *et al.* 2012; Bai *et al.* 2013; Hu *et al.* 2016).

Increases in soil N availability typically enhance performance of invasives relative to natives (Huenneke *et al.* 1990; Daehler 2003; Vinton and Goergen 2006; Qing *et al.* 2011; Mattingly and Reynolds 2014; but see also Lowe *et al.* 2002, Thomsen *et al.* 2006). Exotic plant species are often faster-growing with a higher demand for N (e.g. MacKown *et al.* 2009), and consequently are able to deplete N more rapidly than native plant species that are slower-growing and adapted to N-poor soils (Vallano *et al.* 2012). Nitrification rates have also often been shown to increase in soils associated with exotic plant species (Hawkes *et al.* 2005; Rodrigues *et al.* 2015; Shannon-Firestone *et al.* 2015).

Soil N enrichment not only favours plant species and ecotypes able to respond to elevated N levels but may also shift the soil microbial community in ways that further benefit establishment and growth of exotic plant species (Kulmatiski *et al.* 2006). Soils fertilized with N may exhibit overall declines in bacterial and fungal biomass (Farrer *et al.* 2013; but see Bardgett *et al.* 1999) and declines in the abundance of AM fungi (Treseder 2004; Aleklett and Wallander 2012; Leff *et al.* 2015), as well as shifts in microbial community composition (Bardgett *et al.* 1999; Farrer *et al.* 2013; Dean *et al.* 2014; Leff *et al.* 2015) or function (Johnson *et al.* 1997). Communities of arbuscular mycorrhizal (AM) fungi also shift in response to N fertilization (Johnson 1993; Hawkes *et al.* 2006; Egerton-Warbuton *et al.* 2007; Leff *et al.* 2015), resulting in a less diverse community that may be less beneficial to at least some plant hosts (Johnson 1993; Egerton-Warbuton *et al.* 2007). To the extent to which native plant species depend on particular groups of bacteria or fungi for establishment and growth, and exotic species do not (van der Putten *et al.* 2007*a*), shifts in microbial communities as a result of elevated N may enhance the performance of exotic plant species at the expense of natives and accelerate the pace of plant invasions (Sigüenza *et al.* 2006*a*; Larios and Suding 2014).

A soil microbial community contains both natural enemies of and beneficial organisms for plants. Consequently, whether the microbial community provides a net benefit or harm may depend on the plant species in question (Bever 2002). Rare plant species appear more likely than common plant species to be harmed by their native soil microbial community, suggesting that their rarity may be influenced by their susceptibility to soil parasites and pathogens (Klironomos 2002; Mangan *et al.* 2010). Because natural enemies are thought to be more likely to exhibit host specificity than mutualists (Richardson *et al.* 2000; Mitchell *et al.* 2006), exotic plant species are expected to escape at least some natural enemies while still benefiting from most mutualisms when grown with a native (i.e. foreign to the exotic species) soil microbial community (van der Putten *et al.* 2007*b*). Consequently, for dominant native and exotic plant species, the native soil microbial community should tend to be beneficial (Klironomos 2002; Callaway *et al.* 2004*a*; Reinhart and Callaway 2004; Mitchell *et al.* 2006; Emam *et al.* 2014). However, studies of exotic plant performance in native and sterilized native soil have typically observed greater growth of the exotic species in sterilized soil, suggesting a negative effect of the native soil microbial community on exotic plant species (Callaway *et al.* 2004*b*; Meiman *et al.* 2006; Vogelsang and Bever 2009; but see Sun and He 2010; Emam *et al.* 2014).

Arbuscular mycorrhizal fungi play an important role in plant growth and have been shown to influence relative abundances of different plant species in communities (van der Heijden *et al.* 1998; Hartnett and Wilson 1999). Differences in benefit of AM fungi to exotic and native plant species have rarely been characterized (Pringle *et al.* 2009; Vogelsang and Bever 2009). Exotic plant species appear to be less likely to benefit from AM fungal colonization than native plant species (Klironomos 2003; Pringle *et al.* 2009; Bunn *et al.* 2015; but see Zhang *et al.* 2010), but also less likely than native plant species to exhibit a negative growth response to colonization (Klironomos 2003; Bunn *et al.* 2015).

Exotic plant invasions and elevated soil N are threats to native grasslands throughout the temperate regions of the world. We focused our study on tallgrass prairie, because it is threatened by anthropogenic increases in N availability through deposition and encroachment of industrialized agriculture (Galloway *et al.* 2004) and its persistence depends on our ability to successfully restore degraded prairie lands. At present, perhaps 13 % of the approximately 64 000 000 hectares of former tallgrass prairie in North America remains, primarily as remnants too small to support the full complement of tallgrass species (Samson *et al.* 2004). Despite the importance of both elevated soil N availability and the soil microbial community for understanding the performance of native and exotic plant species, and the likelihood that the two act synergistically to determine competitive outcomes between native and exotic species (Suding *et al.* 2013), very few studies have examined the interactions between soil microbes and soil N availability in evaluating the relative performance of native and exotic species (Sigüenza *et al.* 2006*a*; Larios and Suding 2014). Larios and Suding (2014) found a differential response to soil microbial community of native and invasive plant species that depended on N and competitor.

We conducted a greenhouse experiment to assess the strength of interspecific competition between native and exotic plant species relative to intraspecific competition when grown under natural and elevated levels of N availability and in the presence and absence of the native soil microbial community. We expected intraspecific competition to be stronger than interspecific competition for the exotic plant species and vice versa for each native. We expected the exotic plant species to respond positively and the two native plant species to respond negatively to elevated soil N. We anticipated no difference in performance of the exotic species between soil inoculated with a prairie soil microbial community and uninoculated soil, while the native plant species would be negatively impacted by the loss of microbial associates. Due to the shift in microbial communities that results from N fertilization, we hypothesized that the benefit of the native soil microbial community would disappear in soils enriched in N.

## Methods

### Soils and seeds

Soils were collected from abandoned pasture at Concordia College’s Long Lake Field Station (Long Lake; 46°49'09"N, 95°53'50"W) and from native tallgrass prairie at Pednor State Wildlife Management Area (Pednor Prairie; 47°02'42.9"N, 96°02'12.6"W), both in Becker County, MN, USA, in Fall 2012. Soils were sieved through 1-cm^2^ mesh to remove rocks and debris, and the Long Lake and half of the Pednor Prairie soils were autoclaved damp at 121 °C for 40 min, allowed to rest for 24 h, and reautoclaved to sterilize. The remaining Pednor Prairie soil was reserved for use as living inoculum.

Seeds of the native forb *Oligoneuron rigidum* (stiff goldenrod) were collected from Pednor Prairie and of the exotic forb *Carduus acanthoides* (spiny plumeless thistle) from restored prairie at Long Lake in October 2012; seeds of the native grass *Andropogon gerardii* (big bluestem) were purchased from Prairie Moon Nursery, Winona, MN (plants will be referred to by genus hereafter). *Oligoneuron* and *Andropogon* are both common native species in local remnants of tallgrass prairie with widespread distributions in grasslands in the USA, and *Carduus* is a common invader regionally in prairie restoration sites (Aldrich-Wolfe, pers. obs.). All seeds were stored at 4 °C for 1 month prior to the experiment to break dormancy.

### Nitrogen treatments

**Figure 1:**
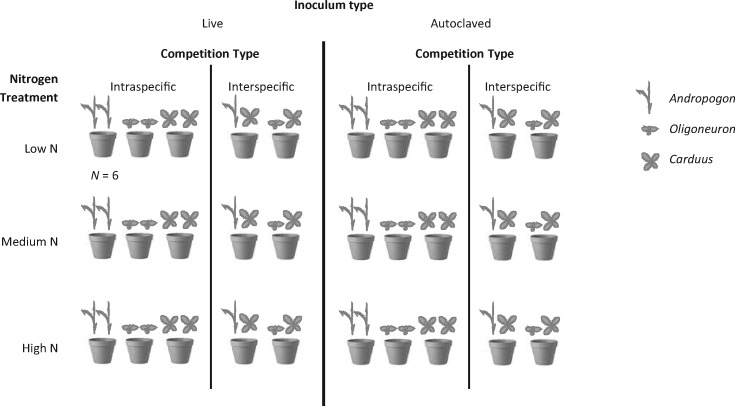
Experimental design. Each of three plant species (the native grass *Andropogon gerardii*, native forb *Oligoneuron rigidum* and exotic forb *Carduus acanthoides*) was grown in intraspecific competition, and each native species was grown in interspecific competition with the exotic species, in pots of autoclaved soil inoculated with living or autoclaved soil from a native prairie and either amended with nitrogen (as NH_4_NO_3_; medium and high N) or not (low N), for a total of 360 plants (180 pots).

Sixty litres of autoclaved Long Lake soil was divided into three equal portions. To prepare the high N treatment, 10 g of NH_4_NO_3_ was added to 20 L of the autoclaved soil; for the medium N treatment, 5 g of NH_4_NO_3_ was added; for the low N treatment, no NH_4_NO_3_ was added. Each soil was then mixed thoroughly and three replicates of 300 mL per treatment were sent to the North Dakota State University Soils Lab (Fargo, North Dakota) for nutrient analysis. Plastic pots (approximately 500 mL in volume) were each filled with 200 mL of soil from one of the N treatments for a total of 60 pots per N treatment and 180 pots overall (Fig. 1; an additional 150 mL of soil were added during soil inoculation, described below).

To maintain differences in N levels in the three treatments, 4 weeks after planting 30 mL of NH_4_NO_3_ solution, at concentrations of 1.25 mg mL^−^^1^ and 2.5 mg mL^−^^1^, were added to each of the medium and high N treatment pots, providing half as much NH_4_NO_3_ as was added to the soil initially. As a control, 30 mL of tap water was added to each of the low N treatment pots. All pots received one-fourth strength complete fertilizer after 7 weeks of growth.

### Inoculum treatments

Fifty millilitres of Pednor Prairie soil were added to half of the low, medium and high N treatment pots (30 pots per treatment) as soil inoculum. As a control for addition of soil inoculum, 50 mL of autoclaved Pednor soil was added to the remainder of the N treatment pots. Pots were capped with 100 mL of the respective N treatment autoclaved soil, to avoid cross contamination by living inoculum. Three replicate 300-mL samples of live and autoclaved inoculum were sent to the North Dakota State University Soils Lab for nutrient analysis.

### Competition treatments

Each of the three species was grown in intraspecific competition, and the two native species (*Andropogon* and *Oligoneuron*) were each grown in interspecific competition with the exotic (*Carduus*) in all three N treatments (low, medium and high N) and in both soil inocula (living and autoclaved; Fig. 1). In February 2013, seeds were planted in two corners on the diagonal (catty-corner, approximately 12 cm apart) in each pot, with 6–10 seeds of a single species per corner, for six pots for each combination of competition type, soil inoculum and N level. The pots were randomized on the greenhouse bench in trays of 10 and watered twice daily; pots were re-randomized biweekly throughout the experiment to minimize bench effects.

### Seedling emergence, survival and growth

Germination success was determined by recording presence or absence of emergent seedlings in each pot corner daily. Both *Carduus* and *Andropogon* had 100 % seedling emergence by week 2. Two weeks after planting, any pots that showed no emergence for *Oligoneuron* were re-seeded. At two weeks, any pot corner with more than two seedlings of *Carduus* or *Andropogon* was thinned to two seedlings; *Oligoneuron* did not require thinning; pot corners that had shown insufficient emergence (0–1 seedling) received a transplant from the replicate pots that had more than two seedlings per corner. Pots were thinned to one seedling per corner after three weeks. At 4 and 15 weeks, seedling survival was recorded.

Plants were harvested after 15 weeks of growth. The aboveground portion of each plant was dried at 98 °C to constant weight to determine aboveground biomass. Roots were washed free of soil, and fine roots < 1 mm in diameter were collected haphazardly from the root ball and stored in 1 % (*w*/*v*) KOH at 4 °C.

### Root colonization by AM fungi

Due to the difficulty of disentangling the root ball for conspecific roots, root samples were pooled within a pot for conspecific plants. Three samples were analyzed for root colonization for each treatment combination in autoclaved soil, and all six samples were analyzed for each treatment combination in living soil. The roots previously stored in 1 % (*w/v*) KOH were rinsed with tap water, acidified in 5 % (*v/v*) HCl for 1 min, and stained with 0.05 % (*w/v*) trypan blue in lactoglycerol (modified from Grace and Stribley 1991). Ten fine roots from each plant were cut into 1-cm pieces, mounted onto a microscope slide with 1:1:1 (*v/v/v*) polyvinyl-lactic acid–glycerol and scored for the presence of AM fungi using a modified gridline intersect method (Giovannetti and Mosse 1980; McGonigle *et al.* 1990) at 400× magnification. The presence of AM fungal structures (hyphae, vesicles, arbuscules) was scored for 100 intersections per slide when sample size permitted (for 21 % of root samples, fewer than 100 intersections were available).

### Statistical analyses

The effects of N level, soil inoculum and competition on emergence and early survival of seedlings were determined by nominal logistic regression separately for *Andropogon* and *Oligoneuron*; no analysis was conducted for *Carduus*, since seedling emergence and survival were 100 % for this species. Effects of N level, soil inoculum and competition on plant biomass and root colonization by AM fungi were analyzed separately for each species by three-way ANOVA after log transformation of biomass and arcsine square root transformation of root colonization to meet model assumptions. For those seedlings of *Oligoneuron* which were too small to weigh accurately, biomass was considered to be zero for statistical analysis. Due to very low survival of *Oligoneuron* plants in high N, the high N treatment was excluded from the ANOVA for *Oligoneuron*. Effect sizes for biomass were estimated as *ω^2^*. The relationship between root colonization by AM fungi and biomass was determined by ANCOVA, taking N level and competition type into account and excluding pots receiving autoclaved inoculum. All statistical analyses were conducted in JMP® v.10 (SAS Institute 2012).

## Results

### Efficacy of treatment establishment

Amending soils with NH_4_NO_3_ and autoclaving soils altered N availability among treatments. Available soil N, both as nitrate and as ammonium, was higher in the high N than in the low N treatment (Table 1; NO_3_-N: *F*_2,12_ = 225.52; *P* < 0.0001; NH_4_-N: *F*_2,12_ = 883.53; *P* < 0.0001). Addition of NH_4_NO_3_ nearly doubled the availability of nitrate between the low and medium N soils, but did not change nitrate availability between medium and high N treatments. In contrast, ammonium availability differed between all three N treatments, nearly doubling from the low to medium N treatments and almost tripling in availability between the low and high N treatments (as intended in the experimental design). While there was no effect of autoclaving on soil nitrate availability (*F*_1,12_ = 0.0002; *P* = 0.99), soil ammonium availability was higher in autoclaved soil than in autoclaved soil amended with live soil at each level of N treatment (Table 1; *F*_1,12_ = 187.13; *P* < 0.0001).

**Table 1.** Nitrogen availability in unamended Long Lake soil (low N), and the same soil after fertilization with ammonium nitrate (medium N and high N), with addition of either living or autoclaved Pednor soil inoculum. Values are means ± SE (*N* = 3), calculated by multiplying means for Long Lake and Pednor soils by their proportions in each pot. Means that share a letter did not differ statistically at α = 0.05 by two-way ANOVA followed by Tukey HSD.

Nitrogen treatment	Inoculum type	Available N (ppm)	
		NO_3_-N	NH_4_-N
Low	Live	50 ± 2^a^	06.9 ± 0.2^a^
	Autoclaved	50 ± 2^a^	16.9 ± 0.2^b^
Medium	Live	95 ± 3^b^	17.5 ± 0.7^b^
	Autoclaved	95 ± 3^b^	27.5 ± 1.7^c^
High	Live	96 ± 3^b^	43.5 ± 1.4^d^
	Autoclaved	96 ± 3^b^	53.6 ± 1.4^e^

Autoclaving strongly reduced, but did not completely eliminate, root colonization by AM fungi. Mean root length colonized by AM fungi was 66 ± 2 % for soil with live inoculum (*N* = 113) and 5 ± 0.7 % for soil with autoclaved inoculum (*N* = 103). N amendment and autoclaving also slightly influenced other soil nutrients and decreased pH [**see Supporting Information—Table S1**].

### Seedling emergence

**Figure 2:**
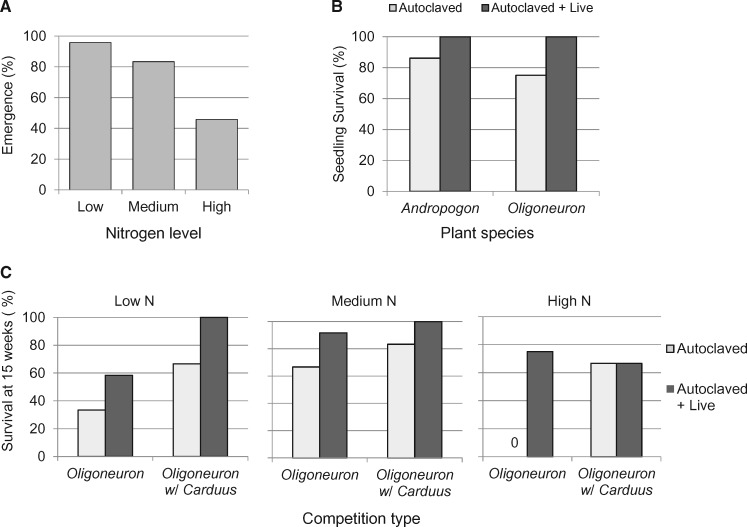
(A) Seedling emergence for *Oligoneuron* at three nitrogen levels (*N *= 24); (B) early survival (≤ 4 weeks) of *Andropogon* and *Oligoneuron* seedlings in autoclaved or living soil inoculum; and C) survival of seedlings of *Oligoneuron* from 4 to 15 weeks of growth in intraspecific competition or interspecific competition with *Carduus* in autoclaved or living inoculum at three N levels (*N* = 6).

Seedling emergence of *Oligoneuron* was negatively affected by N fertilization. Emergence of *Oligoneuron* exceeded 90 % in low N and was below 50 % in high N (Fig. 2A;*χ*^2^ = 17.93, *P* < 0.0001, *DF* = 2). There was no effect of soil microbial community (*χ*^2^ = 0.30, *P* = 0.59, DF = 1) or competition type (*χ*^2^ = 0.00; *P* = 1.00, *DF* = 1) on the emergence of *Oligoneuron* (data not shown). There was no effect of N, soil microbes or competition type on emergence of *Andropogon* or *Carduus*; for both species, emergence was 100 % (data not shown).

### Seedling survival

During the first month of growth, only soil inoculum type had an effect on seedling survival and only for the native plant species. The native soil microbial community had a positive effect on early survival of both *Andropogon* (*χ*^2^ = 5.37, *P* = 0.02, *DF* = 1) and *Oligoneuron* (*χ*^2^ = 10.29, *P* = 0.0013, *DF* = 1); mortality only occurred in pots without living prairie inoculum (Fig. 2B). All *Carduus* seedlings survived from emergence until harvest.

Seedling survival from Weeks 5–15 only differed between treatments for *Oligoneuron* ([**see****Supporting Information**—**Table S3**] for summary; only two seedlings of *Andropogon* died between Weeks 5 and 15). Nitrogen fertilization negatively affected the survival of *Oligoneuron* seedlings (*χ*^2^ = 17.4, *P* = 0.0006, *DF* = 2). Mortality was higher in high N than in low or medium N (Fig. 2C). Inoculation with live prairie soil had a positive effect on survival of *Oligoneuron* (*χ*^2^ = 13.97, *P* = 0.0002, *DF* = 1). Seedlings were far more likely to survive in pots containing living prairie soil inoculum than in pots containing autoclaved inoculum, except when grown with *Carduus* at the highest N level. *Carduus* had a positive effect on survival of *Oligoneuron* (*χ*^2^ = 11.08, *P* = 0.0009, *DF* = 1). *Oligoneuron* seedlings were more likely to survive when grown in competition with *Carduus* than when grown in intraspecific competition (Fig. 2C).

The effect of N fertilization on *Oligoneuron* survival depended on both type of soil inoculum and type of competition (*χ*^2^ = 8.47, *P* = 0.015, *DF* = 2). In low and medium N soils, survival was higher for seedlings grown in competition with *Carduus* than for seedlings grown in intraspecific competition and higher for seedlings inoculated with prairie soil than in autoclaved inoculum (Fig. 2C). In contrast, in high N soil, all *Oligoneuron* seedlings grown in autoclaved soil in intraspecific competition died, while there was no difference in mortality between soil inoculum types for *Oligoneuron* when grown with *Carduus* or between competition types when grown in pots inoculated with prairie soil.

### Seedling biomass

**Figure 3:**
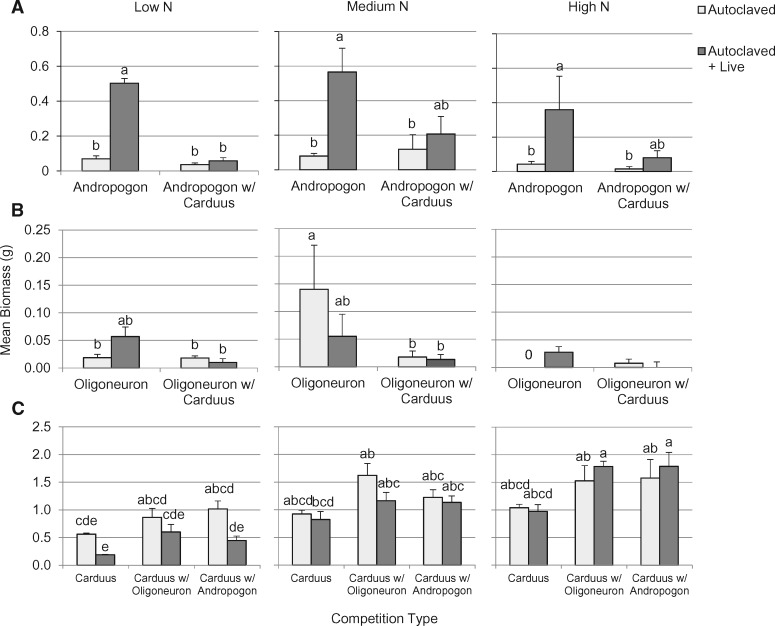
Effects of soil inoculum, N level and neighbour on aboveground biomass after 15 weeks for (A) *Andropogon*; (B) *Oligoneuron*; and (C) *Carduus.* Values are means ± SE (*N *= 6). Means that share a letter did not differ by Tukey HSD. Too few *Oligoneuron* in high N survived to include in statistical analysis. Note differing scales for *y* axes.

N fertilization negatively affected plant growth in the native species and positively affected plant growth in the exotic species. There was a weak negative effect of N level on the biomass of *Andropogon* (Table 2), which grew best in medium N and more poorly in low and high N (Fig. 3A). Mean biomass of *Oligoneuron* was also higher in medium N than in low N (Table 2 and Fig. 3B); high mortality of *Oligoneuron* in the high N treatment precluded statistical comparison with the other two levels. In contrast to the native species, *Carduus* growth increased with increasing N level (Table 2 and Fig. 3C).

**Table 2.** Effects of N level, soil inoculum and neighbour on aboveground biomass after 15 weeks of growth for *Andropogon gerardii* (*N *= 70)*, Oligoneuron rigidum* (*N* = 34) and *Carduus acanthoides* (*N* = 101). ω^2^ is a measure of effect size. The largest effect size for each species and *p* values < 0.05 are shown in bold.

	*Andropogon*				*Oligoneuron*					*Carduus*			
	*DF*	*F*	*P*	*ω^2^*	*DF*	*F*	*P*	*ω^2^*	*DF*	*F*	*P*	*ω^2^*
N level	2	3.07	0.0538	0.03	1	6.04	**0.0209**	0.08	2	44.25	**<0.0001**	**0.45**
Inoculum type	1	32.74	**< 0.0001**	**0.24**	1	1.31	0.2630	0.01	1	6.59	**0.0121**	0.06
Competition type	1	19.88	**< 0.0001**	0.14	1	19.19	**0.0002**	**0.30**	2	17.26	**<0.0001**	0.15
N level × inoculum type	2	0.42	0.6572	0.00	1	5.80	**0.0234**	0.08	2	6.22	**0.0031**	0.07
N level × competition type	2	0.70	0.5022	0.00	1	5.49	**0.0270**	0.08	4	0.31	0.8721	0.00
Inoculum type × competition type	1	16.78	**< 0.0001**	0.12	1	0.37	0.5496	0.00	2	0.18	0.8346	0.00
N level × inoculum type × competition type	2	0.53	0.5913	0.00	1	6.66	**0.0159**	0.09	4	0.86	0.4941	0.00
Error	58				26				83			

The effect of the native soil microbial community on plant growth depended on the plant species. Growth of *Andropogon* was positively affected by soil inoculation (Table 2 and Fig. 3A), while growth of *Oligoneuron* was unaffected by soil inoculum (Table 2 and Fig. 3B). For *Carduus*, there was a small negative effect of soil inoculation on growth (Table 2 and Fig. 2C).

Competition with the exotic plant species had a negative effect on growth of the native plant species. Biomass of *Andropogon* (Fig. 3A) and *Oligoneuron* (Fig. 3B) was greater when they were grown in intraspecific competition than when they were grown in competition with *Carduus* (Table 2). In contrast, *Carduus* biomass was higher when grown in competition with either *Andropogon* or *Oligoneuron* and lower when grown in intraspecific competition (Table 2 and Fig. 3C).

The variable explaining the largest proportion of the variation in biomass differed for each species. For *Andropogon*, the presence of the native microbial community had the largest effect on biomass (Table 2). For *Oligoneuron*, the type of competition explained the most variance, while for *Carduus*, the most important explanatory variable was nitrogen availability.

### Interacting effects of treatments on biomass

The effect of soil N fertilization on *Oligoneuron* growth depended on inoculum and competition type (Table 2). *Oligoneuron* biomass was the highest in autoclaved soil in medium N when grown in intraspecific competition and the lowest in autoclaved soil in low N when grown in intraspecific competition or in either soil inoculum type at either N level in competition with *Carduus* (Fig. 3B).

The effect of soil inoculum type on *Andropogon* growth depended on the type of competition (Table 2). Biomass of *Andropogon* was higher in pots inoculated with living prairie soil than in pots with autoclaved inoculum when grown in intraspecific competition but not when grown in competition with *Carduus* (Fig. 3A).

The effect of soil inoculum on *Carduus* growth depended on N level (Table 2). *Carduus* grew most poorly in low N in soil inoculated with prairie microbes, while there was no difference in biomass between soil inoculum types in medium or high N (Fig. 3C). For *Andropogon* and *Carduus*, there was no interaction between N level, soil inoculum type and type of competition (Table 2).

### Root colonization by AM fungi

All three plant species were extensively colonized by AM fungi in pots inoculated with live prairie soil [**see Supporting Information—Figure S1**]. Soil N level had no effect on root colonization by AM fungi [**see Supporting Information—Table S4**]. There was no effect of competition type on root colonization by AM fungi for either *Andropogon* or *Carduus*. Mean root colonization in live soil inoculum was 74 ± 2 % in *Carduus* (*N* = 52) and 56 ± 3 % in *Andropogon* (*N* = 34). There was a positive effect of being grown with *Carduus* on root colonization by AM fungi in *Oligoneuron* (*F*_1,21_ = 4.976, *P* < 0.0368). Mean root colonization in *Oligoneuron* was 69 ± 5 % when grown with *Carduus (N* = 16) and 50 ± 7 % (*N* = 11) when grown with itself [**see****Supporting Information**—**Figure S1**].

**Figure 4:**
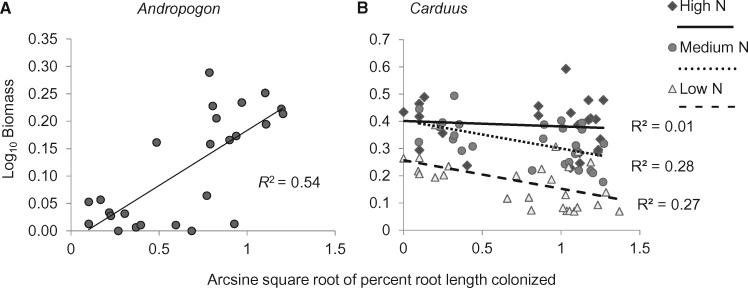
Relationship of aboveground biomass at 15 weeks to root colonization by AM fungi for (A) *Andropogon* when grown in intraspecific competition (*N* = 34); and (B) *Carduus* at low (*N* = 27), medium *(N* = 26) and high (*N* = 23) N levels.

The relationship between root colonization by AM fungi and plant growth depended on plant species and competition type. There was a positive relationship between *Andropogon* biomass and AM fungal root colonization (data not shown; *R*^2^ = 0.31, *P* < 0.0001). The relationship was stronger for *Andropogon* in intraspecific competition (Fig. 4A;*R*^2^ = 0.54, *P* < 0.0001). There was only a weak relationship between root colonization and biomass when *Andropogon* was grown in competition with *Carduus* (data not shown; *R*^2^ = 0.12, *P* = 0.079). For *Carduus*, there was no relationship between root colonization and plant biomass in high N (Fig. 4B;*R*^2^ = 0.01, *P*  = 0.65) and a negative relationship between root colonization by AM fungi and plant biomass in medium N (*R*^2^ = 0.28, *P* = 0.0054) and low N (*R*^2^ = 0.27, *P* = 0.0058). There was no relationship between root colonization by AM fungi and growth in *Oligoneuron* (data not shown; *R*^2^ = 0.02, *P* = 0.404).

## Discussion

The combination of N fertilization, native soil microbes and competition between native and exotic plant species within a single experiment permitted us to measure the relative importance of each factor, as well as to detect important interactions between these variables. In line with our predictions, we found that only the invasive species benefited from increased soil N (Table 3). We found support for the hypothesis that the presence of the native soil microbial community is important for performance of native plant species but not of exotic invaders (Vogelsang and Bever 2009). As expected, *Carduus* grew better with either native plant species than in intraspecific competition, while both native plant species grew better in intraspecific competition. The factor of greatest importance for biomass differed for each plant species: the presence of the native microbe community for *Andropogon*, the absence of *Carduus* for *Oligoneuron* and N fertilization for *Carduus*.

**Table 3.** Summary of effects of nitrogen fertilization, presence of native microbial community and relative strength of intraspecific vs. Interspecific competition on the emergence, survival and growth of the native plant species *Andropogon gerardii* and *Oligoneuron rigidum* and the invasive species *Carduus acanthoides* (↑ positive effect; ↓ negative effect; – no effect; nm, not measured).

	Emergence			Survival			Growth		
Effects	*Andropogon*	*Oligoneuron*	*Carduus*	*Andropogon*	*Oligoneuron*	*Carduus*	*Andropogon*	*Oligoneuron*	*Carduus*
Nitrogen	–	↓	–	–	↓	–	↓	↓	↑
Microbes	–	–	–	↑	↑	–	↑	–	↓
AM fungi	nm	nm	nm	nm	nm	nm	↑	–	↓
Intra- vs. interspecific competition	–	–	–	–	↓	–	↑	↑	↓

For *Andropogon*, there was a large benefit of the native microbial community to growth that was eliminated when grown with *Carduus*. For *Carduus*, there was a negative impact of the native soil community on growth, but only at background levels of soil N availability. For *Oligoneuron*, we observed complex interactions of soil N, the native microbial community and competition type for survival and growth. Emergence and survival were negatively affected by N fertilization. Both the native microbial community and presence of *Carduus* enhanced survival, but their effects were no longer additive at the highest N level. In contrast, N fertilization had a positive effect on growth of *Oligoneuron*, but only in the absence of *Carduus* and the soil microbial community and at intermediate N.

### Effects of nitrogen fertilization on native and invasive plant performance

Increases in N negatively affected seedling emergence and survival of *Oligoneuron*, and growth of both *Andropogon* and *Oligoneuron*, but favoured the growth of the invasive species *Carduus*, suggesting that increased N availability favours establishment of exotic plant species (Alpert *et al.* 2000; Bobbink *et al.* 2010; Littschwager *et al.* 2010; Lee *et al.* 2012; Vallano *et al.* 2012). Because soil ammonium but not nitrate differed between medium and high N treatments, observed differences in plant performance between medium and high N are likely attributable to elevated ammonium availability. Although ammonium availability was nearly three times as high in high N than at ambient levels (low N), elevated levels encountered by the species in this experiment are consistent with values typically observed in regional croplands (Yadav 1997; Bierman *et al.* 2012).

A number of studies have documented a negative relationship between soil N availability and root colonization by AM fungi (Johnson *et al.* 2003; Staddon *et al.* 2004; Treseder 2004; Sigüenza *et al.* 2006*b*). However, we observed no effect of N level on root colonization by AM fungi. Fertilizing soils may select for AM fungi that provide a reduced benefit or increase the carbon cost to the host (Corkidi *et al.* 2002; Johnson 1993). Differences in performance of exotic and native plants between N levels could have resulted in part from differences in the composition and/or function of the AM fungal community, which we did not measure but have been shown to shift in response to N fertilization (Johnson 1993; Egerton-Warburton *et al.* 2007).

Both seedling emergence and survival were negatively affected by elevated N availability for *Oligoneuron*. The few studies that have examined germination or seedling emergence of native and exotic species with varying N availability or for different forms of N observed no effect (Monaco *et al.* 2003; LeJeune *et al.* 2006) or a negative effect of high nitrate on emergence for some native species (Boudell and Stromberg 2015). Ammonium can be toxic to sensitive species, particularly those in soils that naturally contain little N as ammonium (Bobbink *et al.* 2010); susceptibility of native tallgrass species such as *Oligoneuron* to ammonium toxicity and differences in susceptibility between native and exotic species deserve further study.

### Importance of soil microbial community for native and exotic plant performance

In contrast to soil N, which had only weak effects on growth in the native plant species, incorporation of soil inoculum from a local tallgrass prairie had a strong positive effect on growth in the native grass *Andropogon*. This effect is unlikely to be attributable to differences in soil fertility. When differences in nutrient availability were observed between inoculum types (for NH_4_-N and P), nutrient availability was higher in the autoclaved than in the living inoculum. While studies using soils (often repeatedly) cultured by a single host have typically observed negative feedback from the soil to the host (Petermann *et al.* 2008; Perkins and Nowak 2013) for native plant species, studies that have used live inoculum collected below an intact native community (as ours did) have observed a positive growth response of native plant species (Smith *et al.* 1998; Vogelsang and Bever 2009; Larios and Suding 2014; Hilbig and Allen 2015). Repeated culturing of the soil microbial community in pots in a greenhouse likely reduces diversity of the microbial community and may overestimate the importance of pathogenic fungi (Kulmatiski *et al.* 2008).

*Andropogon* has been shown to be highly dependent on colonization by AM fungi in low phosphorus soils such as those used in this study (Hetrick *et al.* 1990; Wilson and Hartnett 1998; Wilson *et al.* 2011). We found root colonization of *Andropogon* by AM fungi to be a good predictor of plant biomass, suggesting that the benefit to *Andropogon* of the presence of the prairie soil microbial community was due in part to the availability of AM associates. While living soil inoculum improved survival for both *Andropogon* and *Oligoneuron*, it did not improve growth in *Oligoneuron*. We observed no relationship between root colonization by AM fungi and biomass for *Oligoneuron*, consistent with the absence of a mycorrhizal growth response observed by Wilson and Hartnett (1998). Consequently, the benefit to this species of the native soil microbial community likely results from the presence of other organisms (e.g. plant-growth-promoting bacteria, archaea, other fungi, protists; van der Putten *et al.* 2007*a*). The high mortality of *Oligoneuron* in the high N treatment (in which ammonium was elevated relative to the medium and low N treatments) in the absence of the native soil microbial community suggests that soil microbes play an important role in mitigating the negative effects of elevated N on seedling survival for this plant species.

A number of studies have documented only a weak association of exotic plant species with soil mutualists such as AM fungi when compared with associations between soil mutualists and native plant species (Sigüenza *et al.* 2006*b*; Pringle *et al.* 2009; Vogelsang and Bever 2009; Jordan *et al.* 2012). In contrast, we found the exotic species to be more heavily colonized by AM fungi than either of the native species. Similarly, Lekberg *et al.* (2013) observed greater colonization by AM fungi of two exotic invasive forbs than of native grass species. However, we found no evidence of a benefit to *Carduus* from colonization. Root colonization by AM fungi was either uncorrelated or weakly negatively correlated with *Carduus* biomass, depending on the N level, suggesting that the difference in performance of *Carduus* between inocula might be due not only to differences in nutrient availability but also to a cost to *Carduus* of associating with AM fungi at least in low N soils. Depending on the host, AM fungi are known to fall along a continuum from mutualism to parasitism (Johnson *et al.* 1997; Klironomos 2003; Reynolds *et al.* 2005; Grman 2012). Callaway *et al.* (2011) observed a weaker benefit or an absence of benefit to exotic plant species of AM fungi from their invasive range relative to AM fungi from their home soils. As a group, exotic plant species appear to be less responsive to colonization by AM fungi than native species (Pringle *et al.* 2009; Bunn *et al.* 2015). There is also some evidence that exotic plant species may evolve to become less dependent on mycorrhizal associations in their invasive range (Seifert *et al.* 2009).

Consistent with the hypothesis that exotic plant species do not benefit from the native soil microbial community (Inderjit and van der Putten 2010; Maron *et al.* 2014), we observed slightly greater growth of *Carduus* in autoclaved than in living inoculum. This difference could be attributable to the greater amounts of ammonium and phosphorus available in the autoclaved inoculum. Because biomass was almost an order of magnitude higher for *Carduus* than for the two natives, *Carduus* was more likely than the natives to have experienced mineral nutrient limitation during this experiment. As observed by Meiman *et al.* (2006), Jordan *et al.* (2008) and Maron *et al.* (2014), our study found no support for the native soil community being more beneficial to an invader than soil in which microbes have been suppressed.

### Relative strengths of intraspecific and interspecific competition for native and invasive plants

Although the outcome of competition did not vary between treatments (*Carduus* consistently emerged as the winner), the performance of *Carduus* was the weakest and the performance of the natives was the strongest in soils that were lower in N and inoculated with living prairie soil. This suggests that success of prairie conservation and restoration will depend on the degree to which soils have been exposed to elevated N and the degree to which the prairie soil microbial community persists following disturbance (Sigüenza *et al.* 2006*a*). Also important is the extent to which native plants and microbes have the capacity to respond evolutionarily to elevated N. In this study, we used local seed of *Oligoneuron* from a native prairie in which N availability is quite low [**see Supporting Information—Table S2**]. Seeds sourced from areas with higher levels of N deposition may produce plants less susceptible to negative impacts from the altered soil microbial community.

For *Andropogon*, competition with *Carduus* effectively eliminated the benefit to the native species of the presence of the prairie soil microbial community. Larios and Suding (2014) observed a benefit to native *Stipa* of its soil community that disappeared when in competition with an exotic. The positive relationship between root colonization by AM fungi and biomass in *Andropogon* was much weaker when *Andropogon* was grown with *Carduus* than when it was grown in intraspecific competition, suggesting that the benefit of the association is diminished by the presence of an invader (Marler *et al.* 1999; Sigüenza et al. 2006*a*; Hilbig and Allen 2015). If exotic plant species form belowground associations with generalist microbes while natives require specific fungal and bacterial associations (Callaway *et al.* 2004*b*; van der Putten *et al.* 2007*b*; Moora *et al.* 2011; Wilson *et al.* 2012), the colonization of natural and restored tallgrass prairies by exotic plant species may alter belowground microbial composition and functionality in ways that further reduce the competitive ability of native plant species (Grman and Suding 2010; Inderjit and van der Putten 2010; Wilson *et al.* 2012; Larios and Suding 2014; Yang *et al.* 2014).

Overall, competition with the exotic species reduced performance of *Oligoneuron*. However, in the high N treatment, survival of *Oligoneuron* seedlings was 0 % in autoclaved soil when grown in intraspecific competition but above 60 % and indistinguishable from survival in living prairie inoculum when grown in competition with *Carduus*. This benefit of *Carduus* to survival of *Oligoneuron* in autoclaved soil, while less dramatic, was also observed at low and medium N and remains to be elucidated. Because *Carduus* grew more vigorously than either native species, soil nutrients would have been drawn down more thoroughly in pots containing *Carduus* than those without. If, as observed for seedling emergence, *Oligoneuron* is negatively affected by soil N availability, perhaps the benefit of *Carduus* derives from the reduction in soil nutrients. The exotic species’ ability to thrive in high N may have mitigated the negative effect of high soil N availability on *Oligoneuron* survival. However, the ability of the exotic to shield the native species from mortality would not benefit the native in the long term since growth of the native was negligible when grown with *Carduus*.

Interactions between native and exotic plants are complex and, while the overall effect of an exotic on a native may be negative, components of the interaction may be beneficial (Lekberg *et al.* 2013). While *Carduus* had a negative effect on growth of *Oligoneuron*, *Carduus* appeared to facilitate survival of *Oligoneuron* in low and medium N soils. Oligoneuron experienced some mortality in all treatments except when grown with *Carduus* in live inoculum in low and medium N, suggesting an inoculum-dependent benefit of *Carduus*. Despite receiving no benefit from colonization by AM fungi, *Carduus* was heavily colonized by AM fungi, and colonization of *Oligoneuron* by AM fungi was substantially higher in live inoculum when *Carduus* was present. These results are consistent with *Carduus* facilitating *Oligoneuron* survival by boosting colonization of *Oligoneuron* by AM fungi, in addition to an apparent direct benefit to *Oligonueron* of a reduction in soil N availability by *Carduus*. Lekberg *et al.* (2013) documented an increase in AM fungal diversity and abundance in association with the invasive plant species *Euphorbia esula*, which also was more heavily colonized by AM fungi than native plant species. In the low and medium N treatments, the effects of the native microbial community and *Carduus* were additive. However, in the highest N level (corresponding to elevated ammonium), the benefit of the native microbial community was only observed in the absence of *Carduus*. This suggests that the protective effect of the native microbial community is lost under elevated N, as also observed for *Stipa* in California grasslands (Larios and Suding 2014).

Our results suggest that prairie managers are wise to consider the negative impact elevated soil N can have on native plant performance, particularly in prairies experiencing exotic invasion. We have assessed the relationships between soil N, the presence of an intact native soil microbial community and competition for three of the many native and exotic plant species currently interacting in plant communities. Studies that examine other native and exotic plant species in a similar experimental framework are clearly warranted to determine the degree to which our results can be extrapolated to native and exotic plant species in general. With the advent of molecular techniques that permit a rapid and comprehensive assessment of community composition belowground, we urgently need more studies that examine the ways in which elevated N and exotic plant species interact to suppress native plant species and which components of the soil microbial community are essential for successful restoration of native prairies.

## Conclusions

We found that background levels of soil N and an intact native soil microbial community are essential to the performance of two native prairie plant species, a grass and a forb, while an invasive forb is most successful under conditions of elevated soil nitrogen and when the native soil microbial community has been disrupted. While other studies have considered either the role of the soil microbial community or the effect of changes in soil fertility on the performance of native and exotic plants, our study is one of the few to evaluate the performance of natives and exotics in relation to both factors and under competitive conditions. This integrated approach allows us to more realistically assess the importance of biotic and abiotic soil factors and their interactions to plant performance and the success of exotic invaders.

## Sources of Funding

This work was supported by the Office of Undergraduate Research and the Biology Department’s Fuglestad-Torstveit Fund at Concordia College.

## Contributions by the authors

Both W.G.S. and L.A.W. designed the experiment, conducted statistical analyses and edited the manuscript. W.G.S. collected all data and wrote the first draft of the manuscript.

## Conflicts of Interest Statement

None declared.

## Supplementary Material

Supplementary Data

## Supporting Information

The following additional information is available in the online version of this article —

**Figure S1.** (A) Mean root colonization by AM fungi ± SE in *Andropogon* and in *Carduus* in autoclaved soil and soil inoculated with the prairie microbial community; and (B) mean root colonization by AM fungi ± SE in *Oligoneuron* in autoclaved soil and soil inoculated with the prairie microbial community when grown in intraspecific competition and in competition with the exotic plant species *Carduus acanthoides*.

**Table S1.** Nutrient concentrations and pH of unamended Long Lake soil and the same soil after fertilization with ammonium nitrate and inoculation with either living or autoclaved Pednor prairie soil.

Description of effects of autoclaving and fertilization on soil nutrient availability and pH.

**Table S2.** Soil characteristics of Pednor prairie soil.

**Table S3.** Effects of N level, soil inoculum and neighbour on survival from Weeks 5 to 15 for *Andropogon gerardii* and *Oligoneuron rigidum*.

**Table S4.** Effects of N level, soil inoculum and neighbour on root colonization by AM fungi after 15 weeks of growth for *Andropogon gerardii*, *Oligoneuron rigidum* and *Carduus acanthoides*.
