# Supplementary material for: Native plants fare better against an introduced competitor with native microbes and lower nitrogen availability
Source: AoB Plants. 2017 Jan 25;9(1):plx004. doi: 10.1093/aobpla/plx004 (PMC5402526; doi:10.1093/aobpla/plx004)
Supplement: Supplementary Data [file plx004_Supp.docx]

Autoclaved

Autoclaved + Live


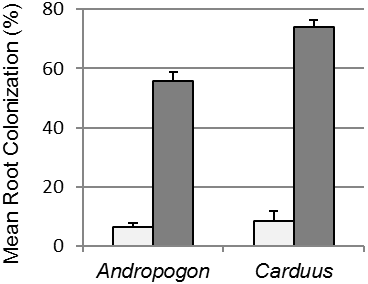

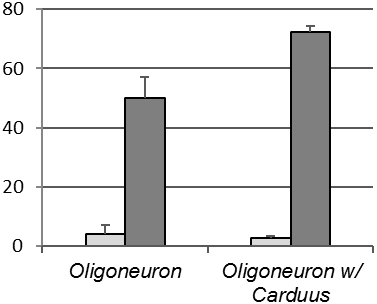


A B B

A

Competition Type

Plant species

**Figure S1. Effect of autoclaving on root colonization by AM fungi.**  A. Mean root colonization by AM fungi (± SE) in *Andropogon* *gerardii* and in *Carduus* *acanthoides* in autoclaved soil (*N* = 17; *N* = 24) and soil inoculated with the prairie microbial community (*N* = 30, *N* = 44); and B. Mean root colonization by AM fungi (± SE) in *Oligoneuron* in autoclaved soil and soil inoculated with the prairie microbial community when grown in intraspecific competition (*N* = 5, *N* = 10) and competition with the exotic plant species *Carduus* (*N* = 7, *N* =15).

**Table S1**. Nutrient concentrations and pH of unamended Long Lake soil (Low N), and the same soil after fertilization with ammonium nitrate (Medium N and High N), with addition of either living or autoclaved Pednor soil inoculum. Values are means + SE (*N* = 3), calculated by multiplying means for Long Lake and Pednor soils by their proportions in each pot. Means that share a letter did not differ statistically at α = 0.05 by two-way ANOVA followed by Tukey HSD.

| **Nitrogen Treatment** | **Inoculum Type** |  |  |  |  |  |  |
| --- | --- | --- | --- | --- | --- | --- | --- |
|  |  | **P** | **K** | **Ca** | **Mg** | **Zn** | **pH** |
| **Low** | **Live** | 7.8 ± 0.3^a^ | 41.3 ± 1.4^a^ | 4265 ± 188 | 231 ± 4^ab^ | 37.2 ± 0.6^a^ | 7.65 ± 0^a^ |
|  | **Autoclaved** | 9.2 ± 0.3^bc^ | 41.0 ± 1.4^a^ | 4348 ± 188 | 219 ± 4^a^ | 37.6 ± 0.6^a^ | 7.69 ± 0^a^ |
| **Medium** | **Live** | 8.9 ± 0.3^ab^ | 49.8 ± 3.8^b^ | 4059 ± 77 | 254 ± 8^b^ | 35.0 ± 0.6^b^ | 7.54 ± 0.03^b^ |
|  | **Autoclaved** | 10.3 ± 0.3^c^ | 49.5 ± 3.8^b^ | 4141 ± 77 | 242 ± 3^ab^ | 35.4 ± 0.6^b^ | 7.57 ± 0.03^b^ |
| **High** | **Live** | 8.9 ± 0.3^ab^ | 49.8 ± 1.4^b^ | 4185 ± 27 | 239 ± 4^ab^ | 28.6 ± 0.7^c^ | 7.60 ± 0.03^ab^ |
|  | **Autoclaved** | 10.3 ± 0.3^c^ | 49.5 ± 1.4^b^ | 4267 ± 27 | 227 ± 4^a^ | 28.9 ± 0.7^c^ | 7.63 ± 0.03^ab^ |

**Effects of nitrogen amendment and autoclaving on soil nutrient availability and pH**

Amendment of soil with NH_4_NO_3_ slightly influenced availability of other soil nutrients and soil pH. Phosphorus availability was low overall but was elevated in the medium and high N treatments relative to the low N treatment (Table 1; *F*_2,12_ = 10.62; *P* = 0.0022). Potassium was lower in availability in the low N treatment than in the medium and high N treatments (*F*_2,12_ = 8.00; *P* = 0.0062), zinc was lowest in availability in the high N treatment and highest in availability in the low N treatment (*F*_2,12_ = 82.68; *P* < 0.0001), and magnesium availability was higher in the medium N treatment than in either the low or high N treatments (*F*_2,12_ = 9.33; *P* = 0.0036). However, all three were still well within the range considered adequate for plant supply in each treatment (http://www.extension.umn.edu/agriculture/nutrient-management). Availability of calcium was unaffected (*F*_2,12_ = 1.55; *P* = 0.2512). As expected, amendment with NH_4_NO_3_ acidified the soil (*F*_2,12_ = 11.77; *P* = 0.0015); pH was highest in the low N treatment and lowest in the high N treatment.

Autoclaving had little effect on soil pH (*F*_1,12_ = 3.19; *P* = 0.0992), but P availability was approximately 10% higher in pots containing autoclaved inoculum than in pots containing live inoculum at each N level (Table 1; *F*_1,12_ = 37.62; *P* < 0.0001). Autoclaving also increased availability of magnesium slightly (*F*_1,12_ = 7.54; *P* = 0.0177). Availability of potassium, calcium, and zinc were unaffected by autoclaving.

**Table S2.** Soil characteristics of unamended Pednor Prairie soil (n = 3).

|  | |  |
| --- | --- | --- |
| Soil character | Mean ± SE | |
|  |  | |
| NO_3_-N | 16.6 ± 1.2 | |
| NH_4_-N | 12.1 ± 0.41 | |
| P | 4.33 ± 0.33 | |
| K | 129 ± 5.9 | |
| Zn | 1.13 ± 0.19 | |
| Ca | 6030 ± 85 | |
| Mg | 840 ± 25 | |
| pH | 7.4 ± 0.03 | |

|  | *Andropogon* | | | *Oligoneuron* | | |
| --- | --- | --- | --- | --- | --- | --- |
| Source | *DF* | *Χ^2^* | *P* | *DF* | *Χ^2^* | *P* |
| N Level | 2 | 0.00 | 1.0000 | 2 | 13.17 | **0.0014** |
| Inoculum Type | 1 | 0.00 | 0.9973 | 1 | 13.97 | **0.0002** |
| Competition Type | 1 | 0.00 | 0.9978 | 1 | 11.08 | **0.0009** |
| N Level × Inoculum Type | 2 | 0.00 | 1.0000 | 2 | 0.00 | 1.0000 |
| N Level × Competition Type | 2 | 0.00 | 1.0000 | 2 | 0.00 | 1.0000 |
| Inoculum Type × Competition Type | 1 | 0.00 | 0.9982 | 1 | 0.00 | 0.9983 |
| N Level × Inoculum Type × Competition Type | 2 | 0.00 | 1.0000 | 2 | 8.47 | **0.0145** |

**Table S3**. Effects of N level, soil inoculum, and neighbor on survival from Weeks 5 to 15 for *Andropogon gerardii* (*N* = 107) and *Oligoneuron rigidum* (*N* = 108)*;* survival in *Carduus acanthoides* (*N* = 144) was 100%.

**Table S4**. Effects of N level, soil inoculum, and neighbor on root colonization by AM fungi after 15 weeks of growth for *Andropogon gerardii* (*N* = 51)*; Oligoneuron rigidum* (*N* = 29)*;* and *Carduus acanthoides* (*N* = 76)*.*

|  | *Andropogon* | | | *Oligoneuron* | | | *Carduus* | | |
| --- | --- | --- | --- | --- | --- | --- | --- | --- | --- |
|  | *DF* | *F* | *P* | *DF* | *F* | *P* | *DF* | *F* | *P* |
| N Level | 2 | 0.21 | 0.8079 | 1 | 2.62 | 0.1202 | 2 | 0.0368 | 0.9639 |
| Inoculum Type | 1 | 142.18 | **< 0.0001** | 1 | 138.86 | **< 0.0001** | 1 | 257.18 | **< 0.0001** |
| Competition Type | 1 | 0.40 | 0.5326 | 1 | 4.97 | **0.0368** | 2 | 1.70 | 0.1910 |
| N Level × Inoculum Type | 2 | 0.28 | 0.7548 | 1 | 0.13 | 0.7216 | 2 | 1.73 | 0.1868 |
| N Level × Competition Type | 2 | 0.72 | 0.4943 | 1 | 1.32 | 0.2639 | 4 | 0.80 | 0.5309 |
| Inoculum Type × Competition Type | 1 | 0.41 | 0.5249 | 1 | 6.80 | **0.0164** | 2 | 0.62 | 0.5403 |
| N Level × Inoculum Type × Competition Type | 2 | 2.11 | 0.1344 | 1 | 2.32 | 0.1424 | 4 | 0.56 | 0.6951 |
| Error | 39 |  |  | 21 |  |  | 58 |  |  |
